# Toward a ‘New Normal’? Tourist Preferences Impact on Hospitality Industry Competitiveness

**DOI:** 10.1057/s41299-021-00123-7

**Published:** 2021-09-08

**Authors:** Maria Teresa Cuomo, Debora Tortora, Alessandro Danovi, Giuseppe Festa, Gerardino Metallo

**Affiliations:** 1 Department of Economics and Statistics, University of Salerno, via Giovanni Paolo II, 132, 84084 Fisciano, SA Italy; 2 Department of Business and Law, University of Milan ‘Bicocca’, Milano, Italy; 3 Department of Business, University of Bergamo, Bergamo, Italy

**Keywords:** Tourist preferences, Tourist expectations, Proximity, Place/destination reputation, Hospitality system competitiveness

## Abstract

The recent outbreak of novel coronavirus (Covid-19) has led to a global panic due to its fatal nature which has harshly impacted the tourist sector and on the place reputation in general. This study aims to compare the factors that develop tourist preferences in terms of (i) what drives the favorability of tourist preferences? (ii) what relationship exists between tourist expectations, proximity, and favorable reputation? and (iii) what are the main influences of tourist preferences on hospitality system competitiveness pre and post Covid-19? By employing structural equation modeling, this study advances knowledge into the research variables’ relationships and advances reputation and marketing performance and practices in the hospitality industry.

## Introduction

The magnitude and severity of the Covid-19 pandemic have dealt a heavy blow to the world travel and tourism sector, with profound economic and social repercussions (Mathieson and Wall 1982; Sigala 2020) on the entire supply chain and on the hospitality system, in particular. Just to have a benchmark, the Covid-19 outbreak impact on the American travel industry in 2020 was about nine times of that from 9/11. Hotel room revenue was cut in half, from $167 billion to $85 billion. Hotels were running at about 44% occupancy in 2020, down from 66% in 2019 (Kwok 2021). Furthermore, the impact of Covid-19 on business travel has varied (from April to December 2020) by region with huge contractions: in North America it declined by 79%; Western Europe 77%; Latin America 59%; Eastern Europe 63%; and Asia Pacific and Middle East and Africa 52% (Stimson 2021). Thus, very deep wounds will probably mark a change of direction in the way the tourism offer is provided (Hall et al. 2020; Gössling et al. 2020). With an overall rethinking, the tourist industry will have to show an unprecedented capacity to serve the changing needs of the tourist, so as to preserve the sector reputation, while at the same time trying to bring out alternative tourist needs (Nientied and Shutina 2020; Wachyuni and Kusumaningrum 2020). Ability to reorganize and reactivate the offer, together with an effective interpretation of the demand (Sigala 2020), will be the new keywords to remain competitive. Will the hospitality industry be able to capitalize in the moment?

Accordingly, the aim of the study is to provide insights that will help hospitality system to understand and interpret new tourist preferences that can build new normality, based on alternative formulas to capture tourists in line with emerging market sensitivities. Considering these arguments and this new context, the current research aims to provide responses to the following queries: (i) Which factors develop tourist preferences? (ii) What drives the favorability of tourist preferences? (iii) Is there a relationship between tourist expectations, proximity, and favorable reputation? and (iv) What are the main influences of tourist preferences on hospitality system competitiveness?

To answer to the abovementioned questions, a conceptual model based on these relationships is developed. To address these relationships, we will use the theory of needs and the theory of demand with variable consumer preferences. Then, the research seeks to examine preferences of tourist about factors that potentially explain expectations, proximity, and reputation and to study whether and how the tourist preferences may influence the hospitality system competitiveness in pre-Covid-19 and during and post-Covid-19 pandemic, using empirical testing of data collected on a sample of 441 tourists in Italy.

The tourism sector represents a perfect scenario for the analysis, due to a sad record: it was the first sector to face the catastrophic and devastating effects of the viral emergency, with evident and current difficulties both in terms of the sector's capacity to maintain and traces of recovery on the outlet markets (Fiavet 2020). It should also be remembered that most of the tourism activities are related to hospitality and, therefore, require contact often—direct and physical—with potential users. That makes difficult to respect the necessary and inevitable ‘social distancing’ practices in the management of the relationship with the virus (Wen et al. 2020) and often brings international visitors to the decision to abandon the trip (for 1 out of 4 tourists, UNWTO 2020). This prerogative of tourism production systems, in this historical phase, is supposed to be a high critical factor, imposing a radical revision of the internal organization and business models for the benefit of workers and tourists (Sigala 2020).

It is always difficult to venture predictions and less than ever in such a picture of uncertainty. Indeed, the countless numbers of forecasts announced in last months by the experts and the press shared a common view: a paralysis of the sector. The most credited Italian estimates, in fact, foresee overall decreases in turnover of almost 30 billion, with an equally significant decrease for the incoming tourism, reduced by 260 million admissions (− 43,4% in 2020 compared to 2019, Cst 2020), with a drop in the connected tourist expenditure of around 4.5 billion (Demoskopika 2020). Depending on the duration of the outbreak, then, the companies in the travel and tourism chain could even double their loss.

In this light, the paper is structured as follows: it starts with an explanation of the conceptual model and presenting a series of hypotheses. Next, the paper sets out the research method. A large-scale field survey investigation is undertaken to examine the results of the research hypotheses. Finally, discussion, implications, and conclusions are presented.

## Theoretical Background and Conceptual Framework

Far beyond analyzing the appropriateness of the interventions—public and private—put in place so far for the support of the tourism sector (which perhaps deserves further study), it is worthwhile to focus the discussion on the responsiveness of the players of the segment to the changes that have occurred (Cillo et al. 2021). It is not yet clear if and when it will be possible to restore the *status quo ante*. However, the tourist offer should adopt a step-by-step approach. Therefore, after the initial moment of the health emergency, to be addressed by trying to resist and limit damages, in the current period of coexistence with the virus, it will be necessary to first manage the emerging needs required by the tourist (i.e., a need for security, Nientied and Shutina 2020). Appropriate reassurance actions will make it possible to recover the trust relationship with the target audience, sometimes limited by crisis information systems and communication (Yu et al. 2020). Only after having stimulating and reorganizing the production of tourist services, it will be possible to proceed with initiatives to stimulate the demand in terms of expectations, preferences, proximity, reputation, and impacts on hospitality system competitiveness (Sukumar et al. 2020). Moving toward the return to normality, it will be necessary to strengthen the tourist offer with renewed sense contents, obviating the age-old problem of overcrowding from mass tourism.

The conceptual model applied in this study is based on two theories. The first one is the theory of needs (Maslow’s hierarchy of needs), while the second is the theory of demand with variable consumer preferences (Basmann 1956). The well-known Maslow theory of needs is considered to define the quality of service as a definition of customers’ needs. This is particularly true in the tourism sector, whereas tourist expectations may be very consistent in the definition of the attributes of the supply (Bi et al. 2020) and in the following definition of preferences.

The theory of demand with variable consumer preferences is based on the fact that individual consumers have no unique ordinal utility index function, that is conversely replaced by a family of ordinal utility functions to be maximized, thus defining advertising elasticity of demand to be satisfied (Chen 2015). In this research, tourist preferences may be considered as a second-order factor, based on inter-correlations among several first-order factors (i.e., tourist expectations, proximity and place/destination reputation). We employed and extended tourist preferences patterns to develop the conceptual model that considers preferences directly affecting vacationer choices in terms of hospitality. The results can be useful to enable managers of the hospitality industry to better understand the competitive positioning of their organizations in the marketplace (Hsu et al. 2009) and to define the strategies and actions able to enhance the competitiveness of the entire system.

**Fig. 1:**
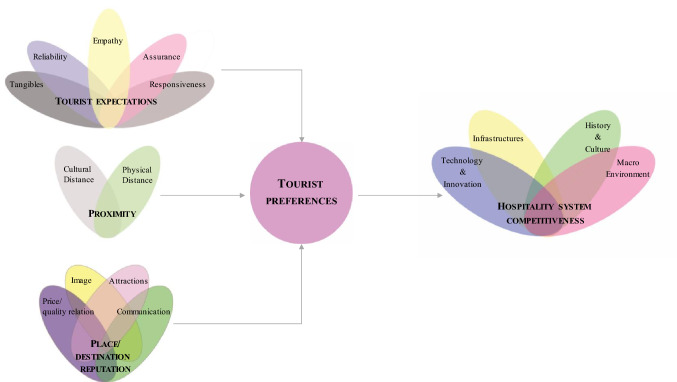
Conceptual framework

Hence, Fig. 1 presents the conceptual model applied in this study.

## Tourist Expectations and Preferences

According to the literature, expectations refer to the aspects, both tangibles and intangibles, that tourists wish or are expected to find in the supply. In that sense, they identify a benchmark to determine customer satisfaction (Pleger Bebko 2000; Tripathi and Siddiqui 2010). Always in line with previous studies (Banerjee and Chua 2016; Dube and Renaghan 1999; Parasuraman et al. 1991; Radojevic et al. 2018), the factors included in the present analysis are retrieved by service quality measurements described by the Servqual scale (Parasuraman et al. 1985; Zeithaml et al. 1990, 1993), and are confirmed as a stable tool for measuring service expectations—and perceptions—across service industries (e.g., hospitality services). According to the Servqual scale, the items to be considered are tangibles, reliability, empathy, assurance, and responsiveness (Parasuraman et al. 1991). Moreover, our analysis will focus exclusively on expectations because the aim of the research is to measure the system of preferences of the hospitality systems, thus overlooking the next post-positivistic model that takes into account three stages of consumer decision processes: pre-purchase influences and decision- making, post-purchase evaluation, and future decision-making (Chen and Gursoy 2001; Moutinho 1987; Mazursky 1989).

Therefore, the firm’s ability to collect and use information about customer needs, called market-sensing capabilities (Likoum et al. 2018), has a positive influence on tourist services planning and need to be constantly increased, in order to intercept future requirements and desires of the demand. This ability to sense and react to the changes of consumer needs and desires, especially linked to crisis events, updating and increasing the value offering, represents a critical factor for maintaining and increasing competitiveness of the hospitality system and the corporate reputation as well (Chun 2005; Kircova and Esen 2018; Pritchard and Wilson 2018).

Based on these considerations, the first hypothesis is as follows:

### H1

The World Tourism Organization (UNWTO)

## Proximity and Tourist Preferences

Carrying on the analysis, the study suggests that tourist preferences are affected by proximity in terms of cultural and physical distance. The first dimension—cultural distance (Boschma 2005; Hofstede 1983, 2001; Rodríguez-Pose 2011; Rutten and Boekema 2012; Torre 2008)—is expressed furthermore as traditions, history, food, etc. of the country of destination; it is very relevant in the assessment of tourism services (Ahn and McKercher 2015; de Carlos et al. 2019; McKercher and Du Cros 2003). The latter—physical distance—, refers to the perceived attractiveness of the destination/accommodation, influenced by a barycentric location, according to tourists planned tours. On this stance, distance does not only represent a physical parameter, but it is related to a psychological and subjective understanding of the tourists’ appreciation of places, perceived as attractive to visit (Jeuring and Haartsen 2017) and accommodations adequate to their standards and desires. Therefore, many tourists consider places near home too familiar and ordinary to satisfy their needs of escape, sense of discovery, searching for exciting experiences associated with being on holiday (Nicolau 2008). In addition, instead of the objectively measured spatial separation, the relational aspects between objects—attractions—across space and their contextualization become meaningful (Larsen and Guiver 2013; Larsen 2015). Thus, the second hypothesis is the following:

### H2

Proximity – in terms of physical and cultural distance – has a positive impact on tourist preferences.

## Reputation and Tourist Preferences

Among numerous definitions of reputation (Fombrun and Shanley 1990; Fombrun 1996; Wagner and Peters 2009; Urde and Greyser 2015), we focus on the tourists' viewpoint. Hence, according to the tourist perception, it can be taken into consideration his/her overall evaluation of a firm, based on his/her reactions to the firm’s products, services, communication activities, interactions (Walsh and Beatty 2007). Then, adapting the concept to a place/destination assessment, we considered its celebrity and offer in terms of attractions to visit or arranged for entertainment, which may influence the price/quality ratio. Moreover, promotional activities dynamically contribute to generating the tourist idea about destination. Consequently, a favorable reputation protects an area and its economic operators/stakeholders against the adverse event, as in health crisis, reassuring vacationers on the engagement of the whole system in making all the proper actions to contrast negative phenomenon (Coafee and Rogers 2008; Cillo et al. 2021). Tourists, on their hand, have a propensity for according a greater trust on such operators compared to destinations with a lower reputation (Foroudi et al. 2016). Hence, investing in a place/destination reputation constitutes a strategy that both public and private partners need to reinforce, as confirmed by the Covid-19 pandemic event. Thus, the third hypothesis is as follows:

### H3

Place reputation has a favorable impact on tourist preferences.

## Tourist Preferences and Hospitality System Competitiveness

Then, we investigated the influence of tourist preferences on hospitality system competitiveness in terms of infrastructures, technology and innovation, history and culture, and macro-environment (Kim et al. 2019). Numerous and well managed public infrastructures (Bahar and Kozak 2007; Bordas 1994; Crouch and Ritchie 1999; Dwyer and Kim 2003; Enright and Newton 2005; Gooroochurn and Sugiyarto 2005; Kozak and Rimmington 1999), make the tourist experience easier, permitting the host to concentrate on the valuable aspects of the vacation. In addition, well-developed technology and innovation have a relevant impact on the tourist experience (Bordas 1994; Chon and Mayer 1995; Gooroochurn and Sugiyarto 2005; Heath 2003). Many studies underlined the unavoidable impact of the development of ICT (Ciampi et al. 2021) on the growing attractiveness of destinations and accommodations, increasingly characterized by intensive information sharing and value co-creation (Akehurst 2009; Porter and Heppelmann 2014; Da Costa Liberato et al. 2018; Stamboulis and Skayannis 2003). Therefore, the culture of sharing and its participatory implications are becoming more and more part of the travel experience for experts and scholars in the sector. Finally, numbers and variety of cultural attractions and places to visit—macro-environment—increase the hospitality system competitiveness (Bordas 1994; Chon and Mayer 1995; Crouch and Ritchie 1999; Dwyer and Kim 2003; Enright and Newton 2005; Sukumar et al. 2020), diversifying the offering in response to the tourist requests and satisfaction (Hong et al. 2020). Hence, we formulated the last hypothesis:

### H4

Tourist preferences have a positive effect on hospitality system competitiveness.

## Methods

### Data Collection

To afford our research questions, we collected data regarding tourists’ perception before and during and post the pandemic crisis. The reason why to choose Italy is the importance of the tourism and hospitality sector, which is one of the key economic drivers of the Country (telegraph.co.uk 2020). However, due to Covid-19, the sector had to face issues globally. The research illustrated that Covid-19 pandemic has significant influences on revenues of the sector by diminution over 40 billion euros, compared to the same period of the earlier year (Statista.com 2020).

To analyze the effects of Covid-19 pandemic on the hospitality system, this study concentrated on the demand for accommodation services based on two main reasons.

(i) The accommodation facilities, initially and still today with great difficulties, had to respond to the changing needs of the tourist, both in that they are not really ready but above all because they had to wait to receive regulatory guidelines and address regarding the methods of providing the services and the time of reopening. This has been confirmed by a sample of hotel structures and territorial tourism development actors who have confirmed the difficulty in responding to potential changes without prior government indications. In this regard, the opening and service protocols have been issued only recently (05-11-2020) connected to the impossibility of moving among Italian regions (06-03-2020). In any case, the analysis of the offer could hardly have made explicit the changes in the expectations of the demand and in the new tourist behaviors (during and post-Covid). On the other hand, the analysis of the demand conducted in the paper has allowed and allows better to bring out the changing needs of the demand in terms of tourist preferences.
(ii) The analysis directly observed the change of attitude of tourists who represent the real actors on which the changes are brought by the pandemic, only as a consequence reversed on the hospitality structures.

We distributed a questionnaire among social media and tourism association in Italy between April and June 2020. We got 473 answers, 441 of which were considered usable. Table 1 illustrates that the sample was composed by a slight majority of female (52.4%) young (born between 1991 to 2000 42.4%); elevated: graduated at secondary school (47.8%), and postgraduate (40.6%). 68.9% of the participants had traveled for vacation around three times during last year (16.1%). 50.6% of the applicants were interested in visiting.

**Table 1:** Participant characteristics

	Frequency	%		Frequency	%
Gender			Recently did you go on vacation		
Female	231	52.4	Yes	137	31.1
Male	210	47.6	No	304	68.9
Age			How many times have you traveled during last year?		
Below 1950	2	0.5	Nothing	243	55.1
Between 1951 to 1960	22	5.0	Once	29	6.6
Between 1961 to 1970	62	14.1	Twice	64	14.5
Between 1971 to 1980	95	21.5	Three times	71	16.1
Between 1981 to 1990	46	10.4	Four times	10	2.3
Between 1991 to 2000	187	42.4	Five times	15	3.4
Below 2000	27	6.1	Six times	5	1.1
Education level			Seven times	3	0.7
PhD			Over seven times	1	0.2
Postgraduate	179	40.6			
Undergraduate	29	6.6			
Secondary school	211	47.8			
Diploma	2	0.5			
Primary school	7	1.6			
What will be the destination of your next vacation?					
Local	12	2.7			
National	223	50.6			
Europe	68	15.4			
Regional	84	19.0			
No vacations	54	12.2			

### Measures

We built the research item measurements according to the literature review and earlier researches. We used six items to measure expectations via five constructs: tangibles, reliability, responsiveness, assurance, and empathy (Banerjee and Chua 2016; Dube and Renaghan 1999; Parasuraman et al. 1991; Radojevic et al. 2018). Proximity was assessed by cultural distance (Boschma 2005; Hofstede 1983, 2001; Rodríguez-Pose 2011; Rutten and Boekema 2012; Torre 2008) and physical distance (Ahn and McKercher 2015; de Carlos et al. 2019; McKercher and Du Cros 2003). The measurement items for reputation were assessed with four items: image, communication, price/quality relation, and attractions (Fombrun and Shanley 1990; Fombrun 1996; Wagner and Peters 2009; Urde and Greyser 2015). Tourist preferences were tested as a single item (Lockyer 2005). In addition, hospitality system competitiveness was expressed with four items: Infrastructure (Bahar and Kozak 2007; Bordas 1994; Crouch and Ritchie 1999; Dwyer and Kim 2003; Enright and Newton 2005; Gooroochurn and Sugiyarto 2005; Kozak and Rimmington 1999), Technology and Innovation (Bordas 1994; Chon and Mayer 1995; Gooroochurn and Sugiyarto 2005; Heath 2003), History and culture (Bahar and Kozak 2007; Crouch and Ritchie 1999; Draper et al. 2011; Dwyer and Kim 2003; Enright and Newton 2005; Go and Govers 2000; Heath 2003; Kozak and Rimmington 1999; Mazanec et al. 2007), and Macro-environment (Bordas 1994; Chon and Mayer 1995; Crouch and Ritchie 1999; Dwyer and Kim 2003; Enright and Newton 2005). Table 2 illustrates the item measurements and references, while the full questionnaire is included in Table 2. We used a seven-point Likert scale(1 = min importance, 7 = max importance).

**Table 2:** Measurement model evaluation for constructs

Constructs	Pre-Covid						Post-Covid					
	Loadings	Mean	StD	*α*	CR	AVE	Loadings	Mean	StD	*α*	CR	AVE
Expectations				0.942	0.954	0.775				0.922	0.940	0.726
Tangibles												
Esthetic care of common areas	0.890	5.628	1.251				0.890	5.628	1.251	Banerjee and Chua (2016), Dube and Renaghan (1999), Parasuraman et al. (1991) and Radojevic et al. (2018)		
Pleasantness/comfort of the room	0.905	5.642	1.246				0.898	5.642	1.246	Banerjee and Chua (2016), Dube and Renaghan (1999), Parasuraman et al. (1991) and Radojevic et al. (2018)		
Reliability												
Attention reserved to any special needs	0.904	5.399	1.341				0.908	5.399	1.341	Banerjee and Chua (2016), Dube and Renaghan (1999), Parasuraman et al. (1991) and Radojevic et al. (2018)		
Responsiveness												
Readiness to respond to any requests	0.879	5.662	1.216				0.879	5.662	1.216	Banerjee and Chua (2016), Dube and Renaghan (1999), Parasuraman et al. (1991) and Radojevic et al. (2018)		
Assurance												
Efficiency/extent of security services	0.851	5.562	1.220				0.660	5.562	1.220	Boschma (2005), Hofstede (1983, 2001), Rodríguez-Pose (2011), Rutten and Boekema (2012) and Torre (2008)		
Empathy												
Friendly climate and atmosphere	0.850	5.431	1.295				0.853	5.179	1.595	Ahn and McKercher (2015), de Carlos et al. (2019) and McKercher and Du Cros (2003)		
Proximity				0.788	0.904	0.825				0.902	0.953	0.911
Cultural distance												
Culture (traditions and customs, history, food, etc.)	0.907	5.755	1.091				0.948	5.386	1.780	Boschma (2005), Hofstede (1983, 2001), Rodríguez-Pose (2011), Rutten and Boekema (2012) and Torre (2008)		
Physical distance												
Proximity of attractions to the accommodation	0.910	5.660	1.145				0.961	5.374	1.704	Ahn and McKercher 2015; de Carlos et al. 2019; McKercher and Du Cros 2003)		
Reputation				0.914	0.940	0.797				0.950	0.964	0.870
Image												
Celebrity/reputation of the place	0.801	5.592	1.222				0.880	5.426	1.500	Fombrun and Shanley (1990), Fombrun (1996), Wagner and Peters (2009), and Urde and Greyser (2015)		
Communication												
Promotional activities on the destination	0.911	5.669	1.224				0.944	5.492	1.496	Fombrun and Shanley (1990), Fombrun (1996), Wagner and Peters (2009), and Urde and Greyser (2015)		
Price/quality relation												
Numbers of entertainment attractions in the area	0.939	5.764	1.228				0.961	5.590	1.508	Fombrun and Shanley (1990), Fombrun (1996), Wagner and Peters (2009), and Urde and Greyser (2015)		
Attractions												
Numbers of places/attractions to visit	0.912	5.708	1.224				0.944	5.537	1.501	Fombrun and Shanley (1990), Fombrun (1996), Wagner and Peters (2009), and Urde and Greyser (2015)		
Preferences				1.000	1.000	1.000				1.000	1.000	1.000
Preferences	1.000	5.685	0.976				1.000	5.685	0.976			
Competitiveness				0.916	0.941	0.799				0.916	0.941	0.799
Infrastructure												
Presence of public infrastructures	0.877	5.352	1.327				0.877	5.352	1.327	Bahar and Kozak (2007), Bordas (1994), Crouch and Ritchie (1999), Dwyer and Kim (2003), Enright and Newton (2005), Gooroochurn and Sugiyarto (2005), Kozak and Rimmington (1999)		
Technology and innovation												
Presence of technological infrastructures	0.920	5.218	1.326				0.920	5.218	1.326	Bordas (1994), Chon and Mayer (1995), Gooroochurn and Sugiyarto (2005) Heath (2003)		
History and culture												
Numbers of cultural attractors	0.901	5.166	1.327				0.901	5.166	1.327	Bahar and Kozak (2007), Crouch and Ritchie (1999), Draper et al. (2011), Dwyer and Kim (2003), Enright and Newton (2005), Go and Govers(2000), Heath (2003), Kozak and Rimmington (1999) Mazanec et al. (2007)		
Macro-environment												
Variety of places/attractions to visit	0.877	5.438	1.236				0.877	5.438	1.236	Bordas (1994), Chon and Mayer (1995), Crouch and Ritchie (1999), Dwyer and Kim (2003), Enright and Newton (2005)		

## Analysis and Model Testing

We examined the research model by using the partial least squares structural equation modeling (PLS-SEM). Based on the number of items together with sample size, PLS-SEM is the better software, as it avoids the constraints of AMOS (Hair et al. 2014). In this study, we employed the measurement and structural models.

### Measurement Model

To examine the reliability and validity, the measurement model was used as a preliminary inspection of the construct’s performance within the entire sample. Cronbach’s *α* and composite reliability were assessed for internal consistency reliability and the items are satisfactory (an *α* and CR above 0.80) (Nunally and Bernstein 1994). Discriminant validity and convergent validity (AVE) were tested for each variable. Table 2 shows that the results of AVEs for variables are above 0.50 (Field 2013). In addition, the indicators’ outer loadings on a construct signifying the discriminant validity is attained (Chin 1998). The results confirmed the respectable reliability of all measures. Table 3 demonstrates the correlations between the research constructs.

**Table 3:** Correlations between constructs

	Tourist expectations	Proximity	Place/destination reputation	TotalCOMP	Tourist preferences
Tourist expectations					
Pre-Covid	1				
Post-Covid	1				
Proximity					
Pre-Covid	0.520**	1			
Post-Covid	0.132**	1			
Place/destination reputation					
Pre-Covid	0.515**	0.421**	1		
Post-Covid	0.411**	0.065	1		
TotalCOMP					
Pre-Covid	0.548**	0.348**	0.564**	1	
Post-Covid	0.541**	0.067	0.407**	1	
Tourist preferences					
Pre-Covid	0.887**	0.670**	0.739**	0.626**	1
Post-Covid	0.873**	0.145**	0.572**	0.626**	1

**The correlation is significant at *p* > 0.01

### Structural Model Assessment

We assessed the structural model results after confirming the construct measures. The collinearity between the constructs was tested before examining the path coefficient assessment. By examining each set of predictors in the structural model for collinearity, each predictor shows the Variance inflation factors (VIF) value was lower than 0.5. Then, we evaluated the significance of path coefficients to explore the hypothesized relationships proposed by the research conceptual model. As Table 4 demonstrates, the importance of the research path coefficients was tested by employing 5000 bootstrapping to create *t*-statistics.

**Table 4:** Path coefficients

	Paths	Expected sign	Pre-Covid					Post-Covid				
			Path coeff	Sample mean (*M*)	Standard deviation (STDEV)	Absolute *t* value	*P* values	Path coeff	Sample mean (*M*)	Standard deviation (STDEV)	Absolute *t* value	*P* values
H1	Expectations → preferences	+	0.600	0.599	0.018	32.867	0.000	0.776	0.775	0.021	36.466	0.000
H2	Proximity → preferences	+	0.217	0.218	0.020	11.112	0.000	0.034	0.032	0.020	1.662	0.097
H3	Reputation → preferences	+	0.337	0.338	0.017	19.704	0.000	0.245	0.247	0.031	7.842	0.000
H4	Preferences → competitiveness	+	0.626	0.626	0.028	22.344	0.000	0.626	0.625	0.027	23.013	0.000

The statistics demonstrated that H1, the impact of tourist expectations on tourist preferences (pre-Covid: *β* = 0.600; post-Covid: *β* = 0.776, *p* < 0.001) was significant from both samples. H2, the impact of proximity on tourist preferences was supported refering to within/pre-COVID (*β* = 0.217, *p* < 0.001); however, the relationships were insignificant refering to post-Covid (*β* = 0.034, p > 0.001). H3 was supported (pre-Covid: *β* = 0.337; post-Covid: *β* = 0.245, *p* < 0.001) and it shows a positive impact of place/destination reputation on tourist preferences. H4 is also supported (Pre-Covid: *β* = 0.626; post-Covid: *β* = 0.626, *p* < 0.001) showing the strong impact of tourist preferences on hospitality system competitiveness.

Lastly, we estimated *R*^2^ values in the path model for the endogenous variables. The *R*^2^ values of our model demonstrated some degree of relationships and clarified over 0.928% of the variances of tourist preferences. To improve the predictive accuracy, we employed Stone-Geisser’s *Q*^2^ value by employing the blindfolding technique for an omission distance of *D* = 7. Hair et al. (2014) stated that the model could be trusted when the predictive relevance of *Q*^2^ is larger than 0. Based on the results illustrated in Table 5, there is a support for the model’s predictive relevance (Chin 1998).

**Table 5:** Results of *R*^2^ and *Q*^2^ values

Endogenous latent variable	Pre-Covid		Post-Covid	
	*R*^2^ value	*Q*^2^ value	*R*^2^ value	*Q*^2^ value
Tourist preferences	0.928	0.922	0.831	0.824
Hospitality system competitiveness	0.392	0.311	0.392	0.311

## Discussions and Implications

Based on the aim of the paper and to minimize the gaps previously underlined, we employed and extended tourist preferences patterns in order to develop our conceptual model (Fig. 1) that considers preferences directly affecting travelers’ decisions in terms of hospitality. The results can be helpful to enable operators of the tourism industry to better interpret the new needs of the marketplace (Hsu et al. 2009) improving the competitiveness of the entire hospitality system.

On this stream, the analysis carried out on pre-Covid and during and post-Covid pandemic is suitable in underlining that when a tourist defines his/her criteria to choose toward lodging, food and drink services, transports, events (Chiang et al. 2019) to attend, and attractions to visit, the first-order factors identified are very consistent and relevant. It is clear the strong tie between tourist expectations and tourist preferences, as demonstrated in H1. In fact, both in pre-Covid and during and post-Covid measurements, the impact of expectations on tourist preferences is observed, indicating that they are scarcely affected by external adverse conditions, e.g., the pandemic event. Hence, the outcomes highlight in terms of theoretical implications that the firm’s ability to collect and act on information about tourist desires has a positive influence on tourist services planning and need to be constantly increased, intercepting future requirements and aspirations of the demand (as widely demonstrated in previous studies: Banerjee and Chua 2016; Dube and Renaghan 1999; Parasuraman et al. 1991; Radojevic et al. 2018). Practically speaking, tourism operators need to really engage in the dialog with customers; social media, for instance, may constitute very interesting tools to directly connect with them (Cuomo et al. 2021).

Moreover, the study hints that tourist preferences are affected by proximity, expressed as a cultural and physical distance in H2. Employing this perspective, we may interpret the results of this research. They show that the impact of proximity on tourist preferences was supported with reference to pre-Covid time. However, the relationships were non-significant when referred to during and post-Covid. This likely expresses a theoretical implication, whereas pandemic outbreak actually has modified the Maslow's hierarchy of needs, ratifying the renewal of safety requirements in terms of personal security and relocating the relevance of proximity—conceived as similarity/closeness instead of distance (Diaz-Soria 2017)—in tourist preferences. However, deeply analyzing the results, it is evident that in the during and post-Covid, the proximity dimension—both in terms of cultural affinity (Hofstede 1983, 2001) and physical closeness of the destination (Ahn and McKercher 2015; McKercher and Du Cros 2003)—can better satisfy the safety needs aforementioned, encouraging tourists to prefer less exotic or faraway destinations (Ahn and McKercher 2015). In this sense, local, regional, or national destinations have been preferred by 72,3% of the sample as a goal of their next vacation, while 12.2% declare they will not go on holidays in 2020. From a practical point of view, this outcome means that closer destinations communicate to the travelers a major sense of control and security, due to a better and easier knowledge toward national procedures and regulations adopted for the progressive resumption of tourism services and for health protocols in Italian hospitality establishments. So, proximity can be considered a ‘new commodity’ and the appreciation of the home region/nation as an appealing form of a tourism destination. In economic and managerial terms, while dramatically changing travel patterns on industry and destinations, Covid-19 crisis creates opportunities for sustainable and proximity tourism (Jeuring and Haartsen 2017; Higgins-Desbiolles et al. 2019; Romagosa 2020). As a matter of fact, home trips may also support a different type of tourism, more respectful of nature and of the visited communities, avoiding mass tourism destinations, where the health danger remains more uncertain (Jamal and Budke 2020). If accurately planned and incentivized, with both public and private support, this contingent variance on the tourism pattern may represent a durable response to the over-tourism phenomenon (Goodwin 2017; Koens et al. 2018; Milano et al. 2018), affecting many Italian cities (the case of Venice, Seraphin et al. 2018), while in the meantime less famous or popular destinations may be proposed as safer places, enjoyable and sustainable from an economic, social, and environmental viewpoint.

These considerations have an impact on the relevance of place/destination reputation (Fombrun 1996; Wagner and Peters 2009; Urde and Greyser 2015) and respect tourist preferences both in pre-Covid and during and post-Covid. Hence, the tourist perception is completely confirmed in H3. Overall evaluation of a firm is based on the reactions to the firm’s goods, services, communication activities, interactions with its representatives, and/or known corporate activities (Walsh and Beatty 2007) in terms of price/quality relations, image, attractions, and communication.

Lastly, we investigated the influence of tourist’s preferences on hospitality system competitiveness, as confirmed in H4 for both pre-Covid and during and post-Covid pandemics. More specifically, innovation, infrastructures, history and culture, and macro-environment improve tourist experience, and they define the most valuable aspects of vacation as described and confirmed by the literature (Gooroochurn and Sugiyarto 2005; Akehurst 2009; Porter and Heppelmann 2014; Da Costa Liberato et al. 2018; Cillo et al. 2021). The theory is confirmed in this case, but the managerial impact needs to understand how it is important to take into account the different variables that impact on tourist’s preferences in order to build strong competitive advantages in the hospitality system.

## Limitations, Future Perspectives of Research, and Conclusions

Despite the interesting results presented above, the study has some limitations. The main limit regards the geographic area of the research process, since the country of origin of the participants under investigation significantly influences the characteristics of the sample, in such case formed by Italian tourists. National culture, system of offering, level of income, etc., deeply affect tourist perceptions and are reflected in the outcomes of the analysis. Thus, to overcome this limit, it would be useful to extend the test to an international sample. Future research, indeed, might compare different clusters of national tourists to evaluate contrasting preferences. The actual sample is also composed mainly by Millennials, in search of unique and authentic experiences, even in the hospitality sector. This generation has less availability of money, but is digital addicted and sensitive toward sustainable issues in the tourism sector, showing greater attention to local communities. However, it would be very compelling to compare the results enlarging the sample to Baby Boomers and Generation X—Covid-19 Generation (Zwanka and Buff 2020). Future studies might analyze the consequences of the hospitality system competitiveness. Furthermore, following studies on possible post-Covid-19 scenarios are essential to help tourism stakeholders profile the offer well, but more accurate data collected on more representative groups are needed.

Finally, the specific period of the analysis needs to be considered. The hospitality industry, and the tourism sector more in general, is facing immense challenges at present, strictly stressed by the global health crisis provoked by the novel Coronavirus–caused respiratory disease Covid-19 (Strielkowski 2020). Even though travel and tourism have been the first economic victims of that situation, at the same time, they have been the principal defendant ‘to sit at the dock’. Since nowadays people move mostly for for tourism reasons, some ascribed to leisure/business movements due to the dissemination all over the world of the Corona outbreak, developed in China last year.

Thus, even though this opinion cannot be shared, the hospitality and travel operators are due to suddenly recover the failure of trust from tourists and local communities. The key lies in the ability to satisfy the surfacing of emergent needs—or perhaps the renewal of old ones on the base of the Maslow Pyramid—linked to safety above all, that have an influence on the effective accessibility and pleasantness of the vacation, affecting the actual touristic demand of hospitality. From now on and waiting for international voyagers come back, the hospitality actors and the public agents need to transform this weakness into an opportunity (Sigala 2020), by investing in the under-tourism and tourism of proximity phenomena—strictly connected to local development (Diaz-Soria 2017)— as the most feasible solutions to answer, in the middle term, the dramatic freeze of the global hospitality offer. For these reasons, it could be interesting investigating on the following topics for the future: no-touch technology anywhere, free cancelations up to 48 h, proximity of high-level hospital facilities, and their impacts on the tourist preferences.

Hence, all stakeholders, including tourists, have a great responsibility, in terms of redirecting tourism, from both supply and demand side, toward a truly sustainable and resilient system, able to answer to future challenges in a more balanced manner, from an economic, social, and environmental viewpoint. This new normal may actually represent a process toward the comprehensive transformation of touristic territories, while always balancing the arrangement of attractive systems of offering, local quality of living, and sustainable development of an area, in terms of favorable repercussions for all the players involved (Uriely et al. 2002). By this way, tourism can be considered as a form of deep civic engagement—more than a simple consumption—favoring the development of a new ethos of sustainable tourism.
